# Temperature Stable Piezoelectric Imprint of Epitaxial Grown PZT for Zero-Bias Driving MEMS Actuator Operation

**DOI:** 10.3390/mi13101705

**Published:** 2022-10-10

**Authors:** Marco Teuschel, Paul Heyes, Samu Horvath, Christian Novotny, Andrea Rusconi Clerici

**Affiliations:** USound GmbH, 1100 Vienna, Austria

**Keywords:** MEMS, speaker, PZT, imprint, bipolar driving

## Abstract

In piezoelectric transducer applications, it is common to use a unipolar operation signal to avoid switching of the polarisation and the resulting nonlinearities of micro-electromechanical systems. However, semi-bipolar or bipolar operation signals have the advantages of less leakage current, lower power consumption and no additional need of a DC−DC converter for low AC driving voltages. This study investigates the potential of using piezoelectric layers with an imprint for stable bipolar operation on the basis of epitaxially grown lead zirconate titanate cantilevers with electrodes made of a metal and metal oxide stack. Due to the manufacturing process, the samples exhibit high crystallinity, rectangular shaped hysteresis and a high piezoelectric response. Furthermore, the piezoelectric layers have an imprint, indicating a strong built-in field, which shifts the polarisation versus electric field hysteresis. To obtain the stability of the imprint, laser doppler vibrometry and switching current measurements were performed at different temperatures, yielding a stable imprinted electric field of −1.83 MV/m up to at least 100 °C. The deflection of the cantilevers was measured with a constant AC driving voltage while varying the DC bias voltage to examine the influence of the imprint under operation, revealing that the same high deflection and low nonlinearities, quantified by the total harmonic distortion, can be maintained down to low bias voltages compared to unipolar operation. These findings demonstrate that a piezoelectric layer with a strong imprint makes it possible to operate with low DC or even zero DC bias, while still providing strong piezoelectric response and linear behaviour.

## 1. Introduction

Piezoelectric transducers have been used for decades for a wide variety of applications, such as energy harvesting [1], micropumps [2], electro-optical modulators [3], sensors [4] and actuators [5]. The expectations of micro-electromechanical systems (MEMS) in terms of cost, linearity and performance are constantly increasing. Due to its superior piezoelectric properties, in particular the strong electromechanical coupling, Pb(Zr_χ_Ti_1−χ_)O_3_ (PZT) is a popular choice for the active layer material [6]. Thin film technologies are needed for submicron-thick PZT layers to enable low operation voltages. The most commonly used deposition technique is the sol-gel process due to its simplicity and low manufacturing costs [7]. However, by depositing PZT layers via a sputtering or pulsed laser process, high crystallinity, low impurity content and defined interfaces can be achieved [8,9]. These two processes allow controlling the stoichiometric properties of the PZT layer by its underlaying layers. The seed layer and the electrode stack on which the piezoelectric layer is grown play an important role for the physical properties such as crystallinity, piezoelectric constant and imprint [10,11]. The imprint is the property that one polarisation state is more likely than the other one; thus, electric fields of different strengths are needed to switch the polarisation from one state to the other. This asymmetric effect is reflected by a shifted polarisation versus electric field (P/E) hysteresis loop. While the mechanism of the imprint is not yet fully understood, it has been attributed to interface effects between the piezoelectric layer and the electrode stack which lead to self-polarisation [12,13]. The literature offers various explanations for the underlying mechanisms of self-polarisation, such as flexoelectricity where a strain gradient induces an electric field [14,15], charge defects between the bottom electrode and the PZT layer [16,17] and asymmetric Schottky barriers created by using different electrode materials or stacks which could lead to an internal electric field [18,19].

The property of an imprint can be useful for MEMS applications, since complicated poling procedures can be avoided [19]. Furthermore, it is usual for MEMS actuators to use a unipolar operation signal by applying a DC bias to circumvent switching of polarisation and hysteresis behaviour, which leads to increased nonlinear behaviour and energy loss [20]. The effect of the imprint allows overcoming these problems due to a shifted hysteresis loop, making semi-bipolar or bipolar operation possible, which is shown in this work. In addition, the temperature stability and benefit of the self-polarisation regarding the operation signal of a MEMS device is explained. For this purpose, the strength of the built-in electric field was determined by the shift of the P/E hysteresis loop. To understand the stability of the imprint, cantilever deflection measurements with a laser doppler vibrometer and switching current measurements were performed at different temperatures. To substantiate the benefit of the imprint, different bias voltages and a constant AC driving signal were applied to the cantilever to see the difference regarding performance. 

## 2. Materials and Methods

For the experiments, MEMS devices with cantilever structures were used. Cantilever structures often form the foundation of MEMS applications, and due to their simplicity, analytical and numerical equations can be used to compare different designs [21]. PZT films of 2 µm thickness were grown epitaxially by sputtering on an electrode stack using silicon as the wafer material. As seed layer yttria-stabilized zirconia was used underneath the bottom electrode stack, consisting of one layer each of platinum and SrRuO_3_ with a total stack thickness of 150 nm. The 200 nm thick top electrode was made of SrRuO_3_. On top of the electrode is an insulation and protective layer stack made of metal oxides, metals and a 45 µm thick polymer layer. Each of the manufactured devices consist of six trapezoidal cantilevers with a total area of 4 mm^2^, which are electrically connected in parallel. Mechanically, they are connected at their tips with a piston (Figure S1 in Supplementary Materials). This design enables the central part of the device to move vertically in Z direction, compared to the bending motion of just a cantilever. The advantage of this specific configuration is to maximise the actuator force, elongation and linearity, chosen especially for audio applications. 

To analyse the switching behaviour, which indicates the strength of the imprint of the piezoelectric material, current measurements were performed using a source meter (2450 SourceMeter, Keithley Instruments, Cleveland, OH, USA). The samples were initially depolarised by a decaying bipolar signal to assure a defined state [22]. For this purpose, a 10 V peak AC signal with a frequency of 1 kHz was applied, and the signal reduced in 1 V steps every second. After the depolarisation, the samples were subjected to a DC voltage that was ramped down from 30 V to −30 V and back up to 30 V again, during which the electric current was measured. The voltage steps were fixed to 250 mV with a delay time of 10 ms between the voltage step and the current acquisition. It should be noted that the switching current amplitudes depend on the measurement delay and the chosen voltage steps. Since the total generated surface charges caused by the dipole switching is constant for a given device and the electrical current is defined as charges per time, the measuring speed changes the measured current amplitude. In addition, if wider voltage steps are chosen, more displacement happens during one step which leads to more charges and results in a higher switching current. For these reasons, the voltage steps and the delay time were kept constant to compare the measurements. The measured electric current is the sum of the displacement current of the dipole switching, the leakage current and the charging current of the capacitor. The leakage current could be neglected due to the high electrical resistance of piezoceramics and the lower applied electric field in comparison to the breakdown field [23,24]. The decaying charging current was visible at the first few voltage steps but became negligible for the voltage region of interest where the dipole switching occurred. To focus on the switching current of the dipoles the charging current was cut off (Figure S2 in Supplementary Materials) [25]. In addition, P/E hysteresis loops were measured with a Sawyer−Tower circuit and an oscilloscope (DSOX1204G, Keysight, Santa Rosa, CA, USA), applying a 30 V peak AC signal at 1 kHz to the setup using a function generator (PSV 500, Polytec, Waldbronn, Germany) in combination with an amplifier (2105 gradient amplifier, AE Techron, Elkhart, IN, USA), in order to determine the imprint [26]. The working principle of the Sawyer−Tower circuit is explained in Supplementary Materials (Section 4). The amplitude of the imprint *E_imprint_* was calculated by the arithmetic mean of the negative coercive field *E_c−_* and the positive coercive field *E_c+_* [27]:

(1) $$E_{imprint}=\frac{E_{c+}-\left|E_{c-}\right|}{2}\;.$$

**Figure 1:**
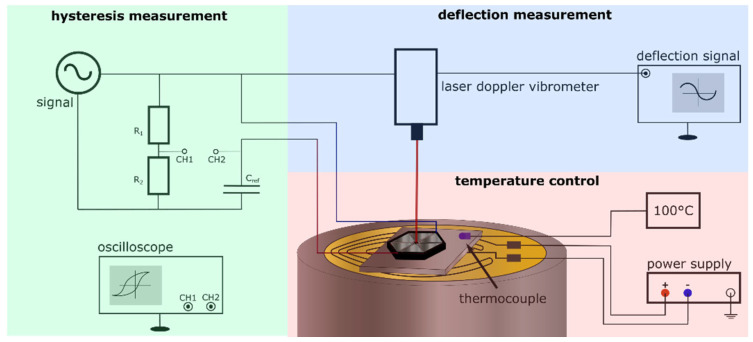
Setup for measuring the polarisation versus the electric field hysteresis loop of the piezoelectric device with a Sawyer−Tower circuit (green). As a measure of performance, the deflection of the MEMS was measured by a laser doppler vibrometer (blue). To obtain the temperature stability of the used devices, a hotplate was heated up by a DC power supply, and the temperature monitored by a thermocouple (red). Micropositioners are not shown for visibility.

In order to assess the temperature stability of the devices, measurements of the hysteresis loops and switching currents were performed at different temperatures. In addition, the piston deflection was used as a measure of performance of the fabricated cantilever structures in this research. The setup used to measure deflection and hysteresis loops under temperature is shown in Figure 1. The MEMS devices were fixed to a hotplate (Polyimide heating film, Thermo Tech, Rohrbach, Germany), with a thin metal plate between the hotplate and the device to ensure homogeneous heating. Micropositioner needles (XYZ 300 TR, Quarter Research & Development, Bend, OR, USA) used to electrically contact the sample provided sufficient pressure to generate good contact between the MEMS and the plate beneath. The hotplate was powered using a DC power supply (PWS4323, Tektronix, Berkshire, UK), while the temperature was measured by a self-sticking thermocouple type k. To evaluate the impact of the temperature on the MEMS performance, piston deflection measurements were performed using a laser doppler vibrometer (PSV 500, Polytec, Waldbronn, Germany), applying a 1 kHz 10 V peak AC signal with a 10 V DC bias for a duration of 10 min at room temperature, at 100 °C and again at room temperature, respectively. Measurements were made in 1 min intervals. To ensure a temperature equilibrium, a 30 min stabilization time was chosen before measurements at each different temperature. The 10 V DC bias was added to prevent any switching from occurring during the measurements, considering the results shown in this work. Switching current measurements were performed using the source meter as described in the previous paragraph under the same three temperature conditions, to analyse the influence of the temperature on the switching behaviour.

To reveal the advantages of a strong imprint regarding possible operating signals, the piston deflection of the MEMS devices was measured at room temperature using a laser doppler vibrometer, applying different DC bias voltages with a constant 10 V peak AC driving voltage at 1 kHz. The applied DC voltage was reduced from 10 V DC to −9 V DC in 1 V intervals. These operation signals revealed the MEMS behaviour at unipolar, semi-bipolar and bipolar actuation. As a quantitative measure of nonlinearity, the total harmonic distortion (THD), which represents the ratio between the sum of displacement of the first five higher harmonics and the displacement of the fundamental actuation frequency, was calculated as:

(2) $$THD_{\%}=\frac{\sqrt{\sum_{n=1}^{5}A_{n}{}^{2}\;}}{A_{0}}\;\cdot 100\;,$$

where *A_n_* are the amplitudes of the higher harmonics of the fundamental actuation frequency and *A*_0_ is the amplitude of the fundamental actuation frequency [28,29]. 

## 3. Results and Discussion

**Figure 2:**
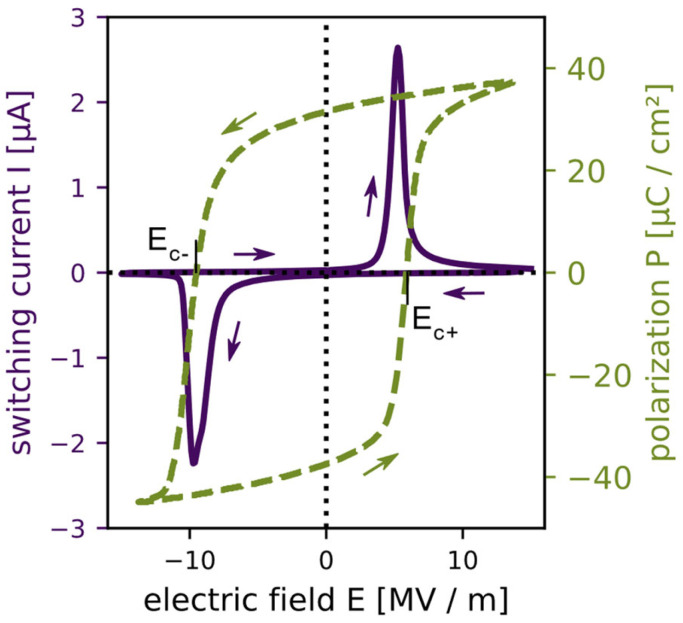
Shifted switching current (solid violet) and polarisation *P* (dashed green) as a function of the applied electrical field *E* to the PZT structures revealing a built-in electrical field, also called an imprint. The measurement direction is indicated by arrows. The strength of the imprint can be calculated by the negative (*E_c−_*) and positive (*E_c+_*) coercive fields.

The bell-shaped switching current and the polarisation hysteresis loop from a device measured at room temperature are shown in Figure 2. Measurements of other devices showed the same characteristics, the results thereof are listed in Supplementary Materials (Sections 3 and 4). By using Equation (1) for the shifted hysteresis loop shown in Figure 2, a built-in field of −1.83 MV/m results. The imprint of the measured switching current can be calculated analogously, where the coercive fields are given by the position of the two current peaks. This gives a value of −2.25 MV/m for the built-in electric field. The calculated imprints are in the range of other reported values [13,14,27]. The mismatch of the coercive fields could be explained by the different characteristics of the presented measurement methods. The hysteresis was measured with a true AC signal with a frequency of 1 kHz, while the switching current was measured quasi-statically by a DC voltage ramp and a delay time between the steps for high current resolution. An investigation in the frequency dependence of the methods showed that the frequency using the Sawyer−Tower circuit had no appreciable influence on the coercive fields between 10 Hz and 1 kHz (Section 6 in Supplementary Materials). This extends the stable range of 15 to 200 Hz reported by Liu et al. [30], based on BaTiO_3_/BTO samples. However, the imprint estimated from the peak positions of the switching current was found to vary by roughly 0.5 MV/m when varying the duration of an entire IV loop between 5 and 1000 s (Section 6 in Supplementary Materials). The reason for this dependency on the measurement speed could lie in the switching dynamics of the measured devices; however, a detailed investigation escapes the scope of this work. Furthermore, the individual switching current peaks display a degree of asymmetry, which affects how accurate the estimation of the coercive field is using the peak position. 

**Figure 3:**
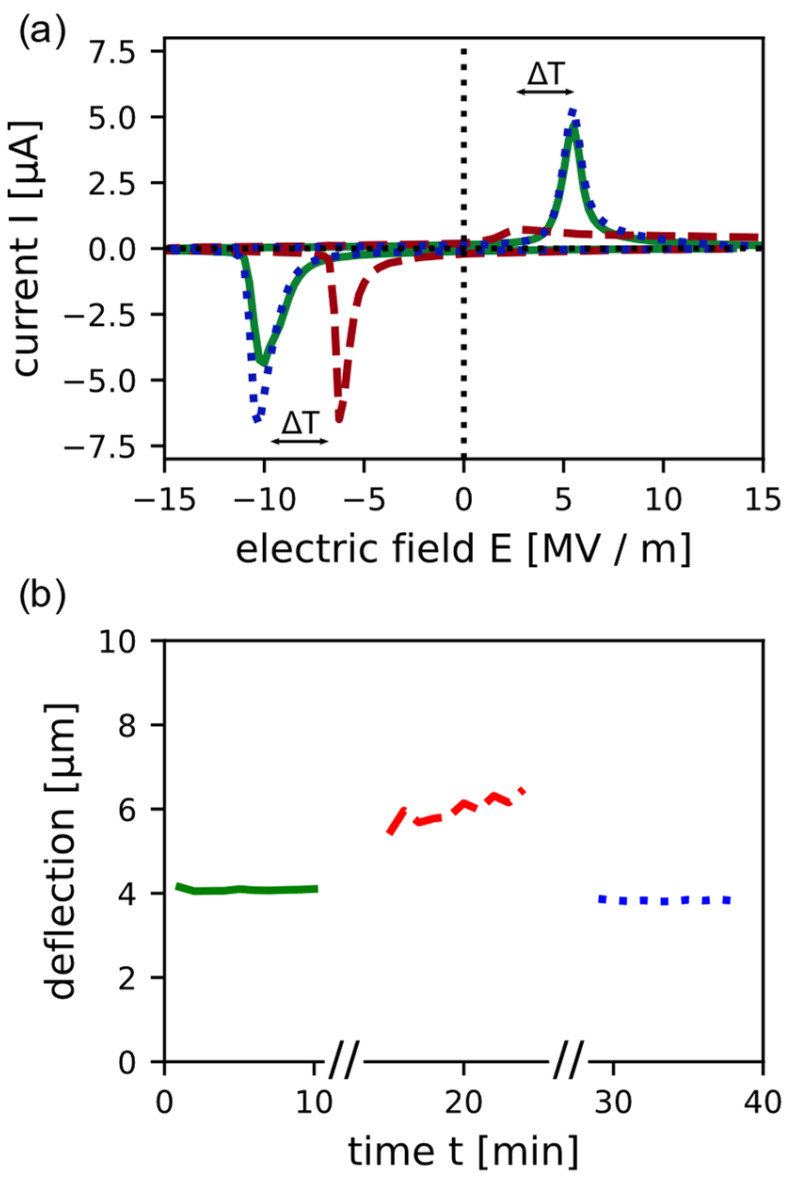
Temperature-dependent switching behaviour of the piezoelectric PZT layer and the deflection of the MEMS device. Measurements at room temperature (solid green line) as the initial, at 100 °C (dashed red line) and at room temperature after heating the sample (dotted blue line) were performed. (**a**) The switching currents as a function of the electric field where a decrease of the imprint at higher temperature is observable; (**b**) the peak deflection before, at and after heating up the sample as a function of time.

The impact the temperature has on the switching behaviour can be seen in Figure 3a, showing the switching currents of a measured device at room temperature, at 100 °C and again at room temperature after exposure to 100 °C. Lower voltages are needed to switch the dipoles at higher temperature compared to room temperature. The imprint reduces from −2.25 MV/m at room temperature to a value of −1.62 MV/m at 100 °C. These results fit to the work of Pintilie et al. [31], where they recorded a decrease of the built-in electric field at higher temperatures. At higher temperatures, the Schottky barrier height decreases which leads to a decrease of the built-in electric field [31,32]. Akkopru-Akgun et al. [27] studied the mechanism and origin of the imprint with Nb-doped and Mn-doped PZT films. They hypothesized that charges from the electrodes are injected and trapped in the interface region due to Schottky emission, which could be the origin of the imprint. This coupling between the Schottky emission and the built-in electric field could be an explanation for the reduction of the imprint at higher temperature in this work. Furthermore, the I/V curve at 100 °C shows that the peak heights of the switching current are asymmetrical. This effect was also reported in the work of Chirila et al. [33], where PZT/SRO/STO/(Si) structures showed an increasing asymmetrical behaviour with increasing temperature. They observed that the dielectric constant has an asymmetric voltage dependency regarding the measuring voltage. This suggests that more free charge carriers are present on one PZT−electrode interface than on the other. These free charge carriers compensate the polarisation charges, which is reflected by a lower switching current [33]. Due to epitaxial growth, the defect density of the bottom interface is lower than the top one, which could cause the asymmetical current peaks [31]. The bell-shaped peak at positive applied field diverges, but the area under the peaks is of the same order of magnitude. Nevertheless, the switching characteristics recover after the high temperature to the same as initially measured, which shows the stability of the imprint. The deflection measurement results shown in Figure 3b agree with the data from the switching behaviour. The peak deflection after the high temperature equals the initial deflection. At 100 °C the peak deflection is higher than at room temperature, which is explained by the softening of the polymeric insulation layer on the cantilevers and the connectors between the cantilever tips and the piston with temperature [34]. The shift of the resonance frequency at different temperatures, which implies a softening of the polymer, is in accordance with the higher deflection with temperature (Section 7 in Supplementary Materials). 

**Figure 4:**
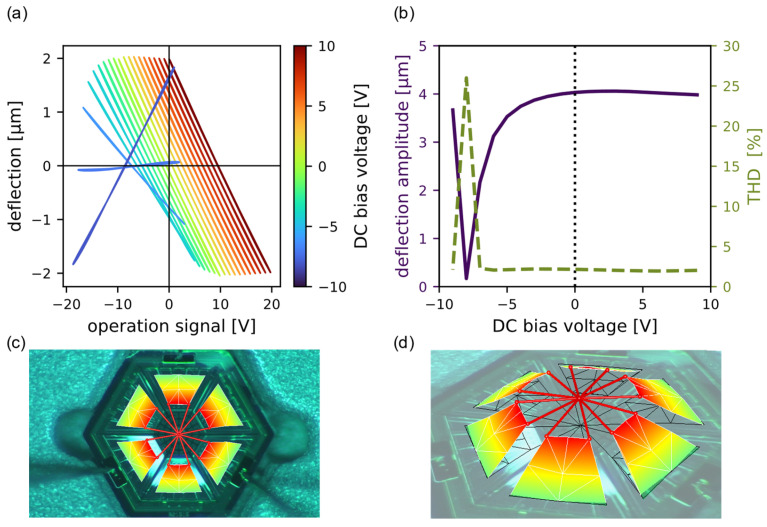
The performance of the MEMS device under different DC bias voltages and a constant applied AC driving voltage: (**a**) the deflection signals at different DC bias voltages; (**b**) the THD (dashed red line) and the peak deflection (solid blue line) as a function of the bias voltages; pictures from the laser doppler vibrometer of the measured MEMS device (**c**) and during operation (**d**).

The piston deflection signals measured at different DC bias voltages are shown in Figure 4a. The nonlinearities start to rise when the applied voltage is in the range of the switching voltage. Outside of the switching voltage range, the applied bias voltage does not have an appreciable influence on the MEMS behaviour. In Figure 4b, the peak-peak deflection amplitude and the THD of the deflection signal, calculated using Equation (2), are shown as a function of the DC bias. At the operation signal of 10 V peak AC and −8 V DC, the negative electric field amplitude −*E_max_* has a value of −8 MV/m, which is in the range of the negative coercive field −*E_c_*. Consequently, the dipoles begin to switch polarisation. When lowering the DC bias past the switching behaviour, the applied voltage signal and the deflection signal are in phase. Figure 4c,d shows the measured MEMS device in the laser doppler setup, where the six trapezoidal cantilevers are connected with a central piston.

In summary, the imprint of fabricated PZT ferroelectric thin films and its temperature stability were studied. Due to the epitaxial growth of the thin films, a built-in electric field of −1.83 MV/m occurred in the ferroelectric layer. The imprint could originate from internal strain-gradients which induce an electric field by the flexoelectric effect, charge defects at the PZT interfaces or from the asymmetric electrodes. Further investigations must be made to distinguish the emergence of the built-in field. Nevertheless, the imprint of the fabricated MEMS devices investigated in this work was found to have a temperature dependence and to recover to its initial value after cooling down. The present study showcases beneficial operation possibilities for MEMS with a strong built-in electric field. With an imprint bipolar, unipolar or semi-bipolar driving signals can be used for actuators without unwanted switching behaviour. By lowering the DC bias voltage, the leakage currents and the power consumption are reduced. In addition, if the amplitude of the driving voltage is lower than the battery voltage of the device, there is no need for additional DC−DC converters to generate the DC bias, which reduces cost and space. 

## Data Availability

If any data is needed the corresponding author can be contacted.

## Supplementary Materials

The following supporting information can be downloaded at: https://www.mdpi.com/article/10.3390/mi13101705/s1. See Supplementary Materials for further information about the fabricated MEMS structure, raw data of the measured switching curves and P/E hysteresis loops of five measured devices, the background of the Sawyer−Tower circuit for measuring the P/E hysteresis loops, further information on the laser doppler vibrometer measurements, the dependence of switching current and Sawyer−Tower measurements on the measurement speed and the temperature dependency of the MEMS resonance frequency. Refs. [22,23,26,34] have been cited in supplementary materials.
