# Supplementary material for: Temperature Stable Piezoelectric Imprint of Epitaxial Grown PZT for Zero-Bias Driving MEMS Actuator Operation
Source: Micromachines (Basel). 2022 Oct 10;13(10):1705. doi: 10.3390/mi13101705 (PMC9606857; doi:10.3390/mi13101705)
Supplement: Supplementary file 1 [file micromachines-13-01705-s001.zip › micromachines-1922326-supplementary.pdf]

SUPPLEMENTARY INFORMATION

Temperature Stable Piezoelectric Imprint of Epitaxial Grown PZT for Zero-Bias Driving MEMS Actuator Operation

Marco Teuschel, Paul Heyes, Samu Horvath, Christian Novotny and Andrea Rusconi Clerici *

USound GmbH, 1100 Vienna, Austria

* Correspondence: andrea.rusconi@usound.com

Table of Contents

1. Supplemental Introduction 3
2. MEMS fabrication and structure 3
3. Switching current measurement 4
4. Sawyer-Tower measurement 6
5. Laser doppler vibrometer measurements 10
6. Influence of measurement speed on IV and P-E loops 11
7. Resonance measurements under temperature 13

1. Supplemental Introduction

The supplementary material gives further information about the measured MEMS structures in this work. Schematically drawn illustrations help to understand where the measuring point for the laser doppler vibrometer measurements was set. In addition an explanation is given for the data post processing of the switching current measurements and the background of the polarization versus electric field P / E hysteresis loop measurement with the Sawyer-Tower circuit. To explain the higher piezoelectric coupling at higher temperatures the results of resonance measurements performed at different temperatures are given.

2. MEMS fabrication and structure

In this work complex MEMS devices were used. Silicon was used as wafer material and the layers were deposited epitaxially by a sputtering process. For the actuators PZT was chosen as piezoelectric material due to its strong piezoelectric coupling. To grow PZT epitaxially the crystal information of the monocrystalline silicon wafer has to be transferred to the piezoelectric layer. For this purpose, the lattice constants of the layers between the wafer and the 2 µm thick PZT layer have to match as well as possible. To achieve the best crystal structure a seed layer made of yttrium-stabilized zirconium YSZ was used. A 45 µm thick polymer layer was deposited on top of the piezo stack as mechanical layer for the cantilever. This polymer also provides insulation from the environment. The manufactured MEMS device is shown

schematically in Fig. S1 and consists of six trapezoidal cantilevers which are connected electrically in parallel. Mechanically the cantilevers are connected to a free moving piston in the middle of the MEMS structure. For the laser doppler vibrometer measurements the laser spot was aligned to the piston to measure the deflection.

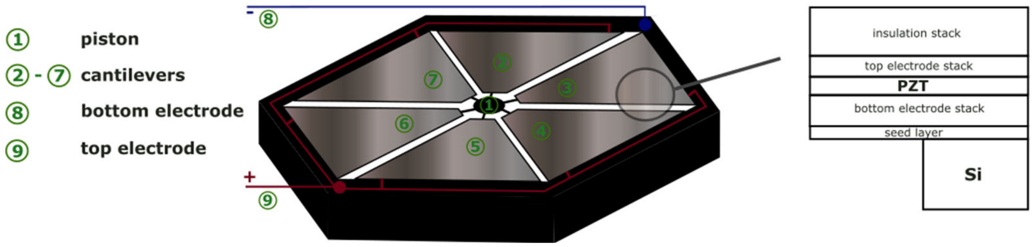

Figure S1. Schematic graphic of the MEMS devices used in this work. The device consists of six trapezoidal cantilevers connected electrically in parallel and mechanically to a central piston.

3. Switching current measurement

The switching current measurements were performed using a source meter (Keithley Instruments, 2450 SourceMeter) ramping down a DC voltage from 30 V to -30 V and back up to 30 V again, while measuring the electrical current. The voltage steps were set constant to 250 mV and the time delay from one voltage step to the next one to 10 ms. Before the measurement the samples where depolarized by a decreasing sinusoidal signal to ensure a defined state.[1] The applied signal started at 10 V peak AC and was reduced by 1 V every second. The measured current consists of the sum of leakage current, switching current of the dipole displacement and charging current of the capacitor-like structure. The leakage current can be neglected due to the high resistivity R[2] of 1 MΩ, measured using a LCR meter (Gw Instek LCR-6100), of PZT. When

the 30 V signal is applied a charging current of a magnitude higher than the switching current is measured. The capacitance C of the devices was measured using the LCR meter and results in a value of 8 nF. To estimate the charging time formula S1,

$$\tau = R \times C , \tag{S1}$$

for a capacitor can be used, where τ is the time constant at which the capacitor is 63.2% fully charged, R is the Resistance and C the capacitance of the device. After 5 τ, which is equivalent to 40 ms for the devices in this work the assumption can be made that the capacitor is fully charged. Fig. S2 shows the switching current as function of the applied electric field of the five measured MEMS devices with the charging current. To focus on the switching current in the main article the charging current was cut off at the data post processing by neglecting the first 5 τ.

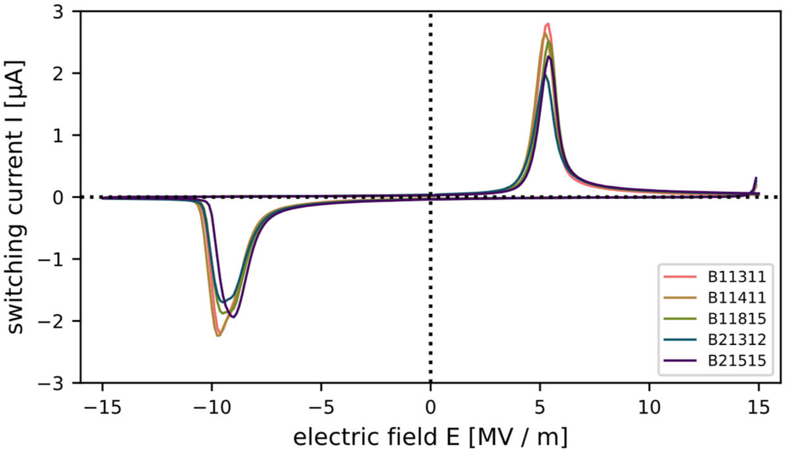

Figure S2. Current as function of the applied electric field of five measured devices. The current is the sum of charging current, leakage current and switching current.

Table S1 shows the relevant settings used to generate the IV curves with the source meter in this work. The largest influence on the result were found to be the settings affecting the measurement speed (see section 6 in the supplementary material). These are the NPLC, step size and delay time, but also setting the range to ‘auto’. Due to the large charging current, the abort condition when exceeding the current limit was switched off.

Table S1. Main source meter settings used to measure the switching current

Source settings	
Range	200 V
Source Readback	On
Measure Settings	
Range	10 µA
Auto Zero	On
NPLC	1
Sense	2-wire
Sweep settings	
Sweep type	Linear Dual
Start / Stop	30 V / -30 V
Step size	250 mV
Source Delay	10 ms
Abort on Limit	Off

4. Sawyer-Tower measurement

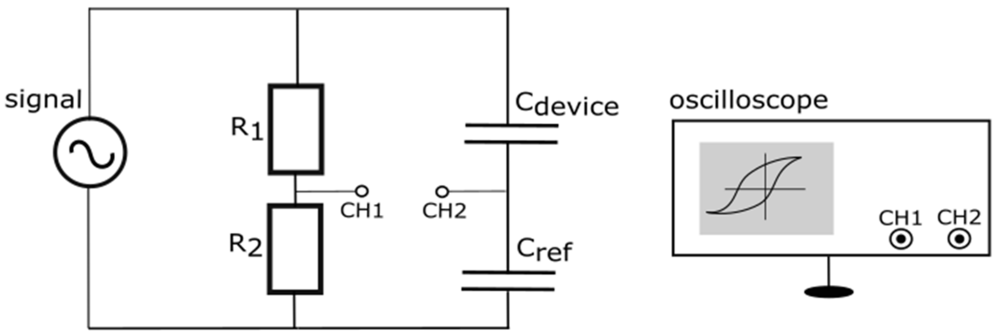

Figure S3. Setup for measuring polarization versus electric field hysteresis loops with a Sawyer-Tower circuit.

To analyse the piezoelectrical behaviour polarization versus electric field hysteresis loops can be used. The graphs provide information about the maximum polarization, coercive field strength, remanent polarization and imprint. To measure hysteresis loops a Sawyer-Tower circuit like in the work of J. Hafner et al.[3] was chosen. The setup shown in Fig. S4 allows to display a proportional hysteresis loop directly on the oscilloscope monitor by measuring voltages with two channels. The measured voltage at channel 1 correlates to the applied electric field on the MEMS device and channel 2 is proportional to the polarization. In the following, the correlation between the measured voltages and the electric field respectively polarization is explained. The voltages applied to the device U_{device} and the reference capacitor U_{ref} result from the equations,

$$U_{device} = \frac{Q_{device}}{C_{device}} \ , \tag{S2}$$

$$U_{ref} = \frac{Q_{ref}}{C_{ref}} \ , \tag{S3}$$

where Q stands for the electric charge and C for the capacitance. Due to the series connection, the charge on the device Q_{device} is equal to the reference charge Q_{ref}. This results in formula S4 from the equations S2 and S3:

$$\frac{U_{device}}{U_{ref}} = \frac{C_{ref}}{C_{device}} \ . \tag{S4}$$

The measured voltage at channel 1 U_{CH1} is measured via a voltage divider with the resistors R_1 and R_2 to ensure low measuring voltages for the oscilloscope. By choosing a reference capacitor at least a magnitude higher than the capacitance of the device $C_{ref} >> C_{device}$ the reference voltage is low enough to ensure no nonlinearities of the reference capacitor occur but still provide enough voltage resolution at channel 2. The measured voltage at channel 1 is given by

$$U_{CH1} = \frac{R_1}{R_1 + R_2} \times (U_{device} + U_{ref})\,. \tag{S5}$$

By including the thickness of the piezoelectric layer d_{PZT} and that the voltage at channel 2 equals the reference voltage a correlation between the measured voltages at channel 1 and 2 of the oscilloscope and the electric field E_{device} applied to the device results:

$$U_{CH1} = \frac{R_1 \times d_{PZT}}{R_1 + R_2} \times (E_{device} + U_{CH2})\,. \tag{S6}$$

The measured voltage at channel 2 is equivalent to the voltage applied to the reference capacitor and can be expressed by formula S4 to:

$$U_{CH2} = U_{ref} = U_{device} \times \frac{C_{device}}{C_{ref}}\,. \tag{S7}$$

By using the equation for a parallel plate capacitor for the device formula S7 can be rewritten,

$$U_{CH2} = \frac{\varepsilon_0 \times \varepsilon_r \times \frac{A}{d_{PZT}}}{C_{ref}} \times E_{device} \times d_{PZT}\,, \tag{S8}$$

with the vacuum permittivity ε_0, relative permittivity ε_r and the total area A of all actuators on the device. By substituting the electric field E_{device} by the polarization P,

$$E_{device} = \frac{P}{\varepsilon_0\,(\varepsilon_r - 1)}\,, \tag{S9}$$

the proportion between the voltage measured with channel 2 and the polarization is given:

$$U_{CH2} = \frac{\varepsilon_r \times A}{C_{ref}\,(\,\varepsilon_r - 1\,)} \times P\,. \tag{S10}$$

By using formula A6 and A10 the P / E hysteresis loop can be calculated by the measured voltages with the oscilloscope. The components of the Sawyer-Tower circuit were set to 10 kΩ for R_1, 1 kΩ for R_2 and the reference capacitor C_{ref} was set to 680 nF which is more than a magnitude higher than C_{device}. The total Area A of all actuators is 4 mm^2 and the relative permittivity ε_r, measured by a LCR meter (Gw Instek LCR-6100), varies between 370 and 470 for the five measured samples. Fig. S4 shows the hysteresis loop of the five measured samples.

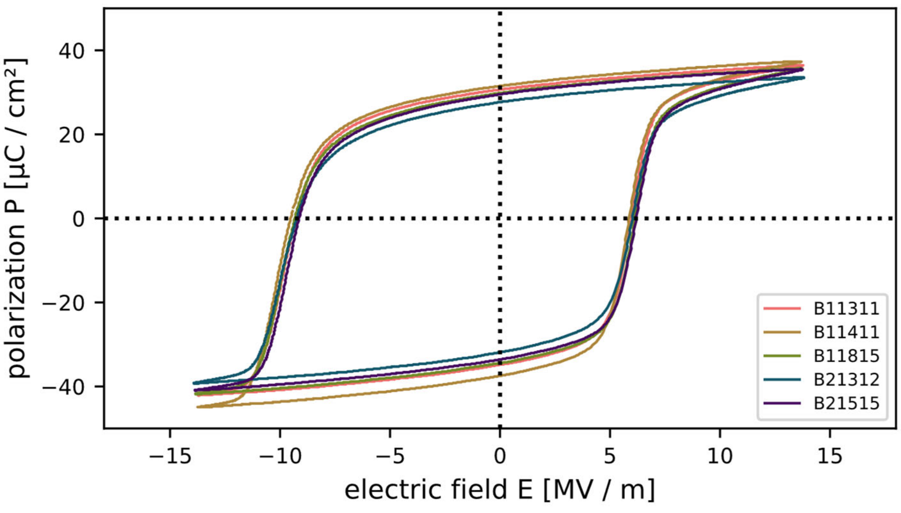

Figure S4. Polarization as function of the applied electric field hysteresis loops of five measured devices.

5. Laser doppler vibrometer measurements

A single point on the piston of the devices was measured in the time domain using a laser doppler vibrometer (Polytec, PSV 500). The internal signal generator of the laser doppler vibrometer (LDV) was used in combination with an amplifier (AE Techron 2105). While the laser doppler vibrometer only measures the velocity signal directly, the measurement software yields the integral, i.e., the deflection. The purely vertical motion of the piston is especially beneficial for this measurement, since there is no loss of laser signal due to reflection of laser light at a large angle, which may be the case for measurements on cantilever tips, for example. All measurements in this work were performed using a 1 kHz sine signal, with various amplitudes and DC bias voltages. A settling time of 1.5 s was chosen after which the signal was recorded. The sample frequency was set to 250 kHz with a measurement duration of 0.1 s. As a result, 100

periods were captured per measurement. In order to obtain the peak-peak deflection, the maximum to minimum value of each period was determined and averaged.

6. Influence of measurement speed on IV and P-E loops

In order to determine the dependence of the calculated imprint on measurement frequency, both presented measurement methods, the IV curves and hysteresis loops, were conducted using different measurement speeds. The hysteresis was obtained at frequencies between 10 Hz and 1 kHz with a 30 V peak signal. For the IV curves the loop duration was varied between roughly 5 and 1000 seconds by altering the current acquisition time, ramping the voltage down from 30 V to -30 V and back up again in 250 mV steps. Both the hysteresis loops and IV curves are shown in Fig. S5, together with the imprint calculated at each individual measurement as a function of measurement loop duration or frequency, respectively.

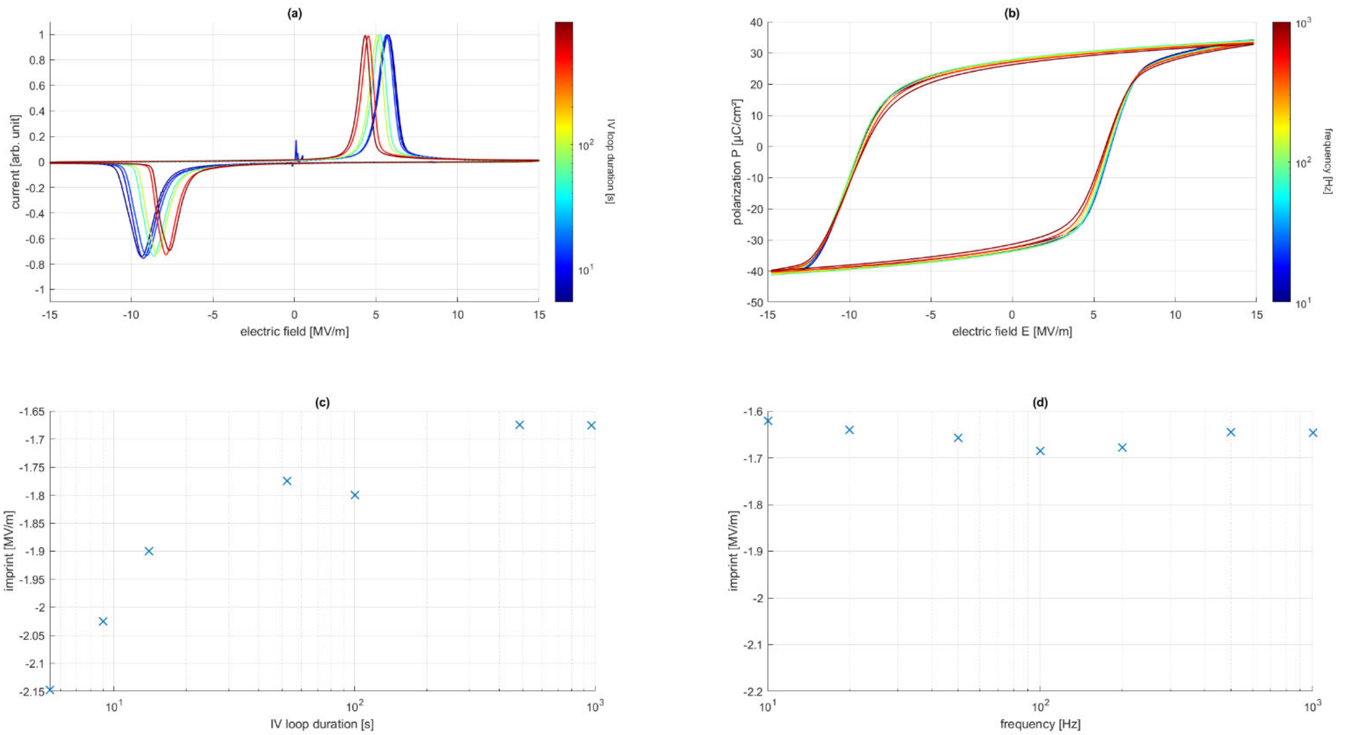

Figure S5. IV curves (a) with associated calculated imprint (c) and hysteresis loops (b) with associated calculated imprint (d) as a function of loop duration and frequency, respectively.

While frequency has no appreciable influence on the coercive fields and consequently the estimated imprint of the P / E measurement, the switching current peak position shows a strong dependence on the IV loop duration, resulting in a difference in imprint of 0.5 MV / m between the slowest and fastest measurement performed here. Compared to the P / E hysteresis, which yields the combined effect of all dipoles, the switching current measurement only captures a fraction of the dipole switching due to its time discrete nature. We assume that the dependence on measurement duration stems from the switching dynamics of different

domains in the material. The resulting switching current is therefore weighted differently over faster or slower domains depending on the measurement speed.

7. Resonance measurements under temperature

To obtain the mechanical temperature behaviour of the MEMS devices, measurements of resonance frequency were performed at different temperatures. The measurements were started at room temperature and repeated while increasing the temperature in 20 °C steps up to 100 °C. The applied voltage was set to 0.1 V peak AC and 10 V DC bias with a frequency sweep from 10 Hz to 80 kHz. Fig. S6 shows the behaviour of the resonance peak with increasing temperature. The resonance frequency decreases with temperature which is attributed to softening of the cantilevers. It is supposed that the main contribution to the softening stems from the 45 µm thick polymeric insulation layer[4]. In addition, the velocity amplitude of the deflection increases with temperature which leads to higher performance of the MEMS with increasing temperature.

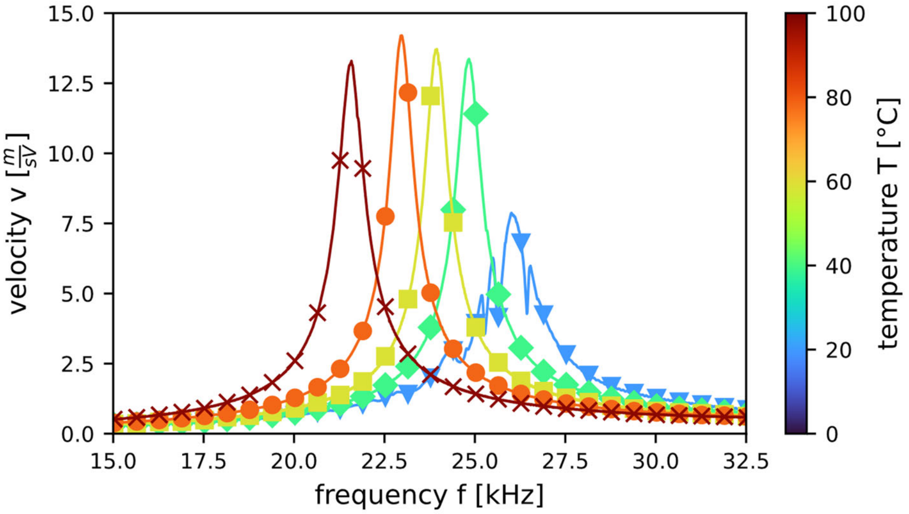

Figure S6. Deflection velocity as function of the frequency at different temperatures. At room temperature (blue triangles), 40°C (green diamonds), 60°C (yellow squares), 80°C (orange circles) and 100°C (red crosses).

REFERENCES

1. Delimova, L.A.; Yuferev, V.S., Transient carrier transport and rearrangement of space charge layers under the bias applied to ferroelectric M/PZT/M structures, *J. Phys. Conf. Ser.* **2019**, *1400*, 055003.
2. Nie, C.; Chen, X.F.; Feng, N.B.; Wang, G.S.; Dong, X.L.; Gu, Y.; He, H.L.; Liu, Y.S., Effect of external fields on the switching current in PZT ferroelectric ceramics, *Solid State Commun.* **2010**, *150*, 101.
3. Hafner, J.; Benaglia, S.; Richheimer, F.; Teuschel, M.; Maier, F.J.; Werner, A.; Wood, S.; Platz, D.; Schneider, M.; Hradil, K.; Castro, F. A.; Garcia, R.; Schmid, U., Multi-scale characterisation of a ferroelectric polymer reveals the emergence of a morphological phase transition driven by temperature, *Nat. Commun.* **2021**, *12*, 152.
4. Tsai, F.Y.; Blanton, T. N.; Harding, D. R.; Chen, S. H., Temperature dependence of the properties of vapor-deposited polyimide, *J. Appl. Phys.* **93**, 3760 (2003)
